# Evaluation of the metabolomic profile through ^1^H-NMR spectroscopy in ewes affected by postpartum hyperketonemia

**DOI:** 10.1038/s41598-022-20371-9

**Published:** 2022-10-01

**Authors:** Anastasia Lisuzzo, Luca Laghi, Filippo Fiore, Kevin Harvatine, Elisa Mazzotta, Vanessa Faillace, Nicoletta Spissu, Chenglin Zhu, Livia Moscati, Enrico Fiore

**Affiliations:** 1 Department of Animal Medicine, Production and Health, University of Padova, Viale dell’Università 16, 35020 Legnaro, Italy; 2 Department of Agricultural and Food Science, University of Bologna, 47521 Cesena, Italy; 3 Department of Veterinary Medicine, University of Sassari, 07100 Sassari, Italy; 4 Department of Animal Science, Pennsylvania State University, State College, PA 16801 USA; 5 Istituto Zooprofilattico Sperimentale delle Venezie, 35020 Legnaro, Italy; 6 College of Food Science and Technology, Southwest Minzu University, Chengdu, Sichuan China; 7 Istituto Zooprofilattico Sperimentale dell’ Umbria e Marche, 06126 Perugia, Italy

**Keywords:** Animal physiology, Diagnostic markers

## Abstract

Ketosis is one of the most important health problems in dairy sheep. The aim of this study was to evaluate the metabolic alterations in hyperketonemic (HYK) ewes. Forty-six adult Sardinian ewes were enrolled between 7 ± 3 days post-partum. Blood samples were collected from the jugular vein using Venosafe tubes containing clot activator from jugular vein after clinical examination. The concentration of β-hydroxybutyrate (BHB) was determined in serum and used to divide ewes into assign ewes into: Non-HYK (serum BHB < 0.80 mmol/L) and HYK (serum BHB ≥ 0.80 mmol/L) groups. Animal data and biochemical parameters of groups were examined with one-way ANOVA, and metabolite differences were tested using a t-test. A robust principal component analysis model and a heatmap were used to highlight common trends among metabolites. Over-representation analysis was performed to investigate metabolic pathways potentially altered in connection with BHB alterations. The metabolomic analysis identified 54 metabolites with 14 different between groups. These metabolites indicate altered ruminal microbial populations and fermentations; an interruption of the tricarboxylic acid cycle; initial lack of glucogenic substrates; mobilization of body reserves; the potential alteration of electron transport chain; influence on urea synthesis; alteration of nervous system, inflammatory response, and immune cell function.

## Introduction

In dairy ruminants, high metabolic demand occur during late gestation and early lactation due to fetal growth and milk production^1^. The majority of the fetus’ growth (from 60 to 80% fetal body weight increase) occurs in the last month of pregnancy^2,3^. Mammary uptake of amino acids, glucose and fatty acids is rapidly increased during early lactation to produce milk^4^. At the same time intake is suppressed and when increased energy demands are not met by increasing intake, animals enter a stete of negative energy balance (NEB) and mobilize body reserves from adipose and muscle tissues^5,6^.

The NEB and tissue mobilization increase the risk of metabolic diseases, especially ketosis. During NEB lipid stores are mobilized and enter the blood as non-esterified fatty acids (NEFA). The liver uptake and metabolism of blood NEFA can result in ketone body production due to a partial oxidation of the fatty acids^7^. Among the ketone bodies, β-hydroxybutyrate (BHB) is commonly used as an indicator of NEB during the last weeks of pregnancy and during the first weeks of lactation^2^. Elevated serum BHB levels, also called hyperketonemia, may leads to a metabolic disease called ovine pregnancy toxemia (OPT) when it occurs in the last 3 to 6 weeks of gestation or lactation ketosis when it occurs in early lactation^3,8,9^. Both of these diseases can occur as clinical or subclinical presentation^10^. Serum BHB ≥ 0.80 mmol/L concentration is the current accepted metric for diagnosis of OPT, but a specific threshold for lactation ketosis is lacking. However, BHB ≥ 0.80 mmol/L is also commonly used for subclinical HYK in sheep during early lactation^3,9^. Furthermore, recent literature in dairy cows reveals that increased BHB concentration (or hyperketonemia) may not be simply related to increased serum NEFA. Greater prepartum BHB is associated with greater postpartum BHB levels and may indicate differences in liver function or other metabolic adaptations^11^. Considering the considerable impact of this disease, preventing hyperketonemia is essential for optimal animal health, welfare, and productivity^11,12^.

Ketosis represents one of the most important health problems in high-producing dairy (cows, sheep and goats)^8^. Major economic losses in ketotic sheep result from death of the affected animals, medication costs, production losses and triggering of secondary diseases^8^. Inadequate energy balance in pregnant sheep is dangerous not only to the dam, but also to pre- and perinatal viability and performance of lambs^1^. Indeed, limited metabolite availability during pregnancy may increase risk of low birth weight and perinatal mortality^1^.

Metabolomics is a newest ‘omics’ science and a powerful tool to elucidating disease etiology, developing biomarkers to detect and characterize diseases as well as to monitor and predict complex diseases^13^. Nowadays, different technologies of metabolomics exist and among them the ^1^H-NMR presents important advantages, including its easy sample preparation and fast results providing high efficiency and reproducibility^14,15^. Furthermore, NMR is inherently quantitative as the signal is proportional to the concentration of the detectable ^1^H nuclei in the receiver coil of the spectrometer, independently from the molecules harboring the nuclei As a consequence, ^1^H-NMR largely employed to determine the purity and stability of standards to be used as reference for other quantification techniques^16^.

In this study, we hypothesized that the development of hyperketonemia in ewes is associated with changes in specific groups of metabolites potentially related to functional mechanism of ketosis. We expected these alterations could be detected using ^1^H-NMR metabolomics and could provide a better explanation of the etiopathogenesis of ketosis. In view of the above considerations, the aim of the current study was to compare the serum of normal and hyperketonemic ewes using ^1^H-NMR to assess the alterations in metabolic profile.

## Results

### Main characteristics

Serum BHB, glucose and urea concentrations was different between the two groups (*p*-value < 0.001, *p*-value = 0.009, and *p*-value = 0.007, respectively). Serum BHB and urea concentrations were greater in HYK compared to the Non-HYK group (BHB mean: 1.35 vs 0.63 mmol/L, SEM = 0.24; Urea mean: 7.66 vs 6.08 mmol/L, SEM = 0.39), whereas glucose concentration was greater in Non-HYK compared to HYK (Glucose mean: 4.07 vs 3.43 mmol/L, SEM = 0.17). However, the two groups did not differ in NEFA (0.17 in Non-HYK vs 0.27 in HYK, SEM = 0.05; mEq/L), parity (3.19 in group Non-HYK vs 2.33 in group HYK, SEM = 1.49), BCS (3.13 in Non-HYK vs 2.61 in HYK, SEM = 0.91), DIM (4.88 in Non-HYK vs 4.08 in HYK, SEM = 0.94; days) or daily milk yield (1.25 in Non-HYK vs 1.22 in HYK, SEM = 0.10; kg/day) (Table 1).

**Table 1:** Mean values ± standard of the mean (SEM) of clinical data and biochemical parameters of dairy ewes divided in group Non-HYK (BHB < 0.80 mmol/L) and group HYK (BHB ≥ 0.80 mmol/L).

Parameters	Group non-HYK (n = 28)	Group HYK (n = 18)	*p-*values^5^
Age (years)	4.60 ± 0.21	4.58 ± 0.20	0.402
Parity	3.19 ± 1.47	2.33 ± 1.50	0.707
BCS^1^	3.13 ± 0.69	2.61 ± 1.13	0.130
DIM^2^	4.88 ± 0.66	4.08 ± 1.22	0.815
Daily milk yield (kg/day)	1.25 ± 0.06	1.22 ± 0.04	0.827
BHB^3^ (mmol/L)	0.63 ± 0.12	1.35 ± 0.35	< 0.001
NEFA^4^ (mEq/L)	0.17 ± 0.04	0.27 ± 0.05	0.146
Glucose (mmol/L)	4.07 ± 0.14	3.43 ± 0.19	0.009
Urea (mmol/L)	6.08 ± 0.33	7.66 ± 0.45	0.007

^1^Body condition score.

^2^Days in milk.

^3^β-Hydroxybutyrate.

^4^Nonesterified fatty acids.

^5^*P*-values corrected with Bonferroni method.

### Serum metabolome profile and robust principal component analysis (rPCA)

**Figure 1:**
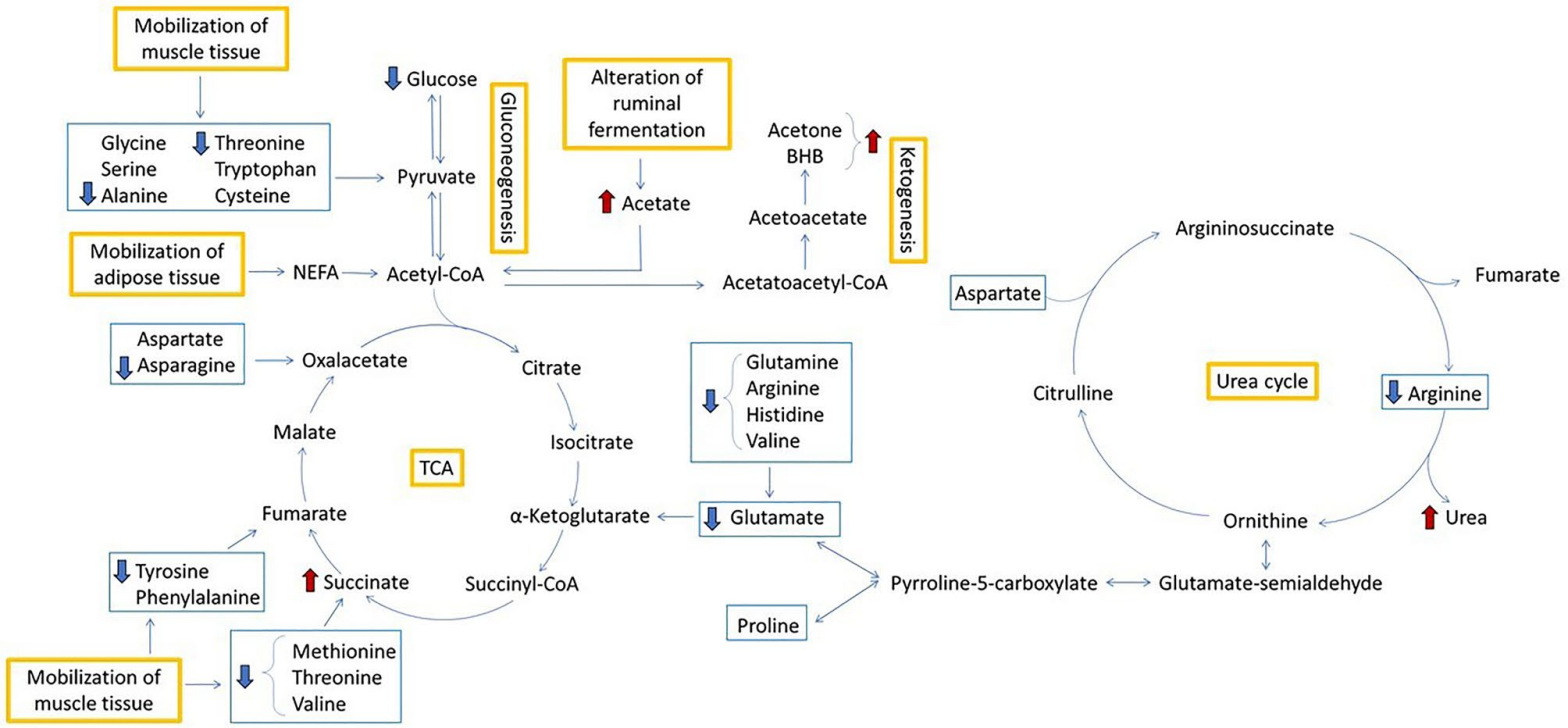
Metabolites changes in group HYP (n = 18; BHB ≥ 0.80 mmol/L) related to tricarboxylic acid cycle (TCA) and urea cycle. Blue rectangles are used to identify amino acids and orange rectangles are used to identify metabolic processes; blue arrows indicate a decrease in metabolite concentration while red arrows indicate an increase in concentration.

**Figure 2:**
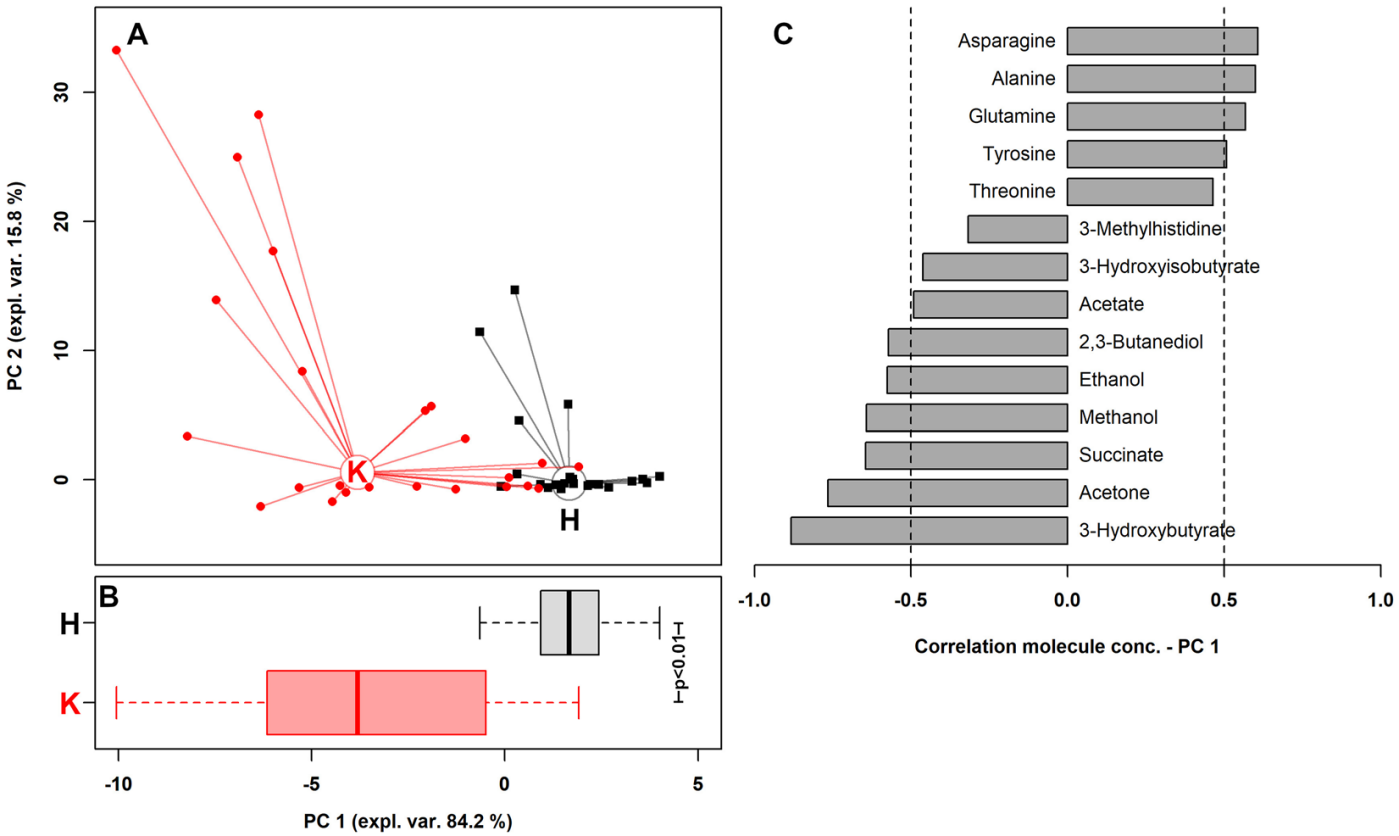
rPCA model built on the space constituted by the concentration of the molecules which showed a statistically significant difference between different groups. In the scoreplot (**A**), samples from the two groups are represented with black squares (Group non-HYK; n = 28; BHB < 0.80 mmol/L) and red circles (Group HYK; n = 18; BHB ≥ 0.80 mmol/L). The wide, empty circles represent the median of each samples’ group. The metabolites distribution along the principal component 1 (PC1) is summarized in the boxplot (**B**). The loading plot (**C**) reports the significant correlation between the concentration of each substance and its importance over PC 1 (p < 0.05).

Fifty-four different metabolites were identified in the serum samples by ^1^H-NMR (Table 2). Among the identified metabolites, fourteen were significantly different between HYK and Non-HYK based on Bonferroni corrected *p*-values (tyrosine, p-value = 0.001; 3-methylhistidine, p-value = 0.015; threonine, *p*-value = 0.013; asparagine, *p*-value < 0.001; glutamine, *p*-value < 0.001; alanine, *p*-value = 0.001; succinate, *p*-value < 0.0001; acetate, *p*-value = 0.025; 3-hydroxyisobutyrate, *p-*value = 0.009; methanol, *p*-value = 0.019; ethanol, *p*-value = 0.008; 2,3-butanediol, *p*-value = 0.002; acetone, *p*-value < 0.0001; and 3-hydroxybutyrate, *p*-value < 0.0001), and five tended to differ (valine, *p*-value = 0.097; glutamate, *p*-value = 0.096; histidine, *p*-value = 0.064; arginine, *p*-value = 0.076; and methionine, *p*-value = 0.095). Metabolites changes related to the tricarboxylic acid cycle (TCA) and urea cycle were represented in Fig. 1. The first principal component (PC1), accounted for 84.2% of all the samples’ variance and summarized the major differences between groups with HYK appearing at low PC1 scores, and Non-HYK, appearing at high PC1 scores (Fig. 2A). The metabolites distribution along the PC1 was summarized in the boxplot (Fig. 2B). The loading plot (Fig. 2C) showed that the most representative metabolites of group HYK were 3-hydroxybutyrate, acetone, succinate, methanol, ethanol, 2,3-butanediol, acetate, 3-hydroxyisobutyrate, and 3-methylhistidine, while the most representative metabolites of group Non-HYK were asparagine, alanine, glutamine, tyrosine, and threonine.

**Table 2:** Mean values and standard error of means (SEM) of identified metabolites (μmol/L) within the two groups (group Non-HYK with BHB < 0.80 mmol/L and group HYK with BHB ≥ 0.80 mmol/L).

Class	Metabolite	Group non-HYK (n = 28)	Group HYK (n = 18)	SEM	*p*-values^2^
Amino acids and derivates	Asparagine	16.00	11.80	0.76	< 0.001
	Glutamine	59.20	47.50	2.00	< 0.001
	Alanine	57.40	48.90	1.78	0.001
	Tyrosine	9.94	7.70	0.44	0.001
	Threonine	36.70	29.80	2.02	0.013
	3-Methylhistidine	10.40	13.40	0.83	0.015
	Histidine	15.80	14.50	0.49	0.064
	Arginine	67.00	58.50	4.99	0.076
	Methionine	4.58	4.09	0.20	0.095
	Glutamate	61.10	55.50	2.47	0.096
	Valine	51.00	43.70	2.61	0.097
	Proline	20.30	18.90	0.76	0.144
	Serine	24.90	23.00	1.89	0.545
	Aspartate	1.63	1.41	0.10	0.257
	Lysine	14.40	14.10	0.84	0.798
	Isoleucine	25.90	25.10	1.29	0.652
	Leucine	49.80	47.70	2.12	0.539
	Dimethylglycine	3.63	4.05	0.24	0.484
	Glycine	125.00	139.00	7.79	0.283
	Betaine	20.90	20.20	1.87	0.637
	Phenylalanine	6.45	5.62	0.27	0.134
	Creatine	39.10	41.60	1.87	0.631
	Creatinine	1.16	1.14	0.10	0.744
	Taurine	24.70	21.20	2.41	0.144
	Sarcosine	0.57	0.54	0.02	0.369
	N6-Acetyl-Lysine	6.76	6.58	0.37	0.572
	2-Aminobutyrate	6.47	6.56	0.28	0.940
Organic acids	Succinate	1.55	2.19	0.11	< 0.0001
	3-Hydroxyisobutyrate	3.60	4.52	0.25	0.009
	Acetate	134.00	170.00	11.55	0.025
	Formate	7.59	8.41	0.52	0.395
	Pyruvate	4.53	3.99	0.25	0.154
	Lactate	283.00	288.00	23.40	0.905
	Citrate	22.60	26.90	2.09	0.283
	Fumarate	0.68	0.76	0.04	0.121
Alcohols	2,3-Butanediol	0.86	2.35	0.31	0.002
	Ethanol	2.12	5.56	0.94	0.008
	Methanol	15.40	48.10	11.15	0.019
	Glycerol	18.40	19.00	2.09	0.889
	myo-Inositol	11.10	12.10	0.80	0.493
Carbohydrates	Glucose	1093.00	1017.00	46.70	0.371
	Mannose	7.03	8.12	0.72	0.388
	Lactose	10.90	11.40	1.86	0.728
Amine and derivates	TMAO^1^	53.70	51.60	4.11	0.456
	Dimethylamine	0.40	0.47	0.03	0.194
Fatty acids	Isovalerate	3.63	4.05	0.24	0.220
	Methylsuccinate	0.57	0.78	0.12	0.358
Ketone bodies	3-Hydroxybutyrate	40.20	103.30	7.34	< 0.0001
	Acetone	6.05	19.52	1.88	< 0.0001
Sulfone	Dimethyl sulfone	10.30	10.30	0.74	0.780
Vitamin	Choline	2.17	2.32	0.25	0.864
Imidazole	Allantoin	10.50	10.30	0.55	0.695
Nucleoside	Uridine	4.05	3.81	0.19	0.492
Guanidine	Methylguanidine	1.02	1.13	0.05	0.103

^1^Trimethylamine-N-oxide.

^2^*P*-values corrected with Bonferroni method.

### Over representation analysis (ORA)

**Figure 3:**
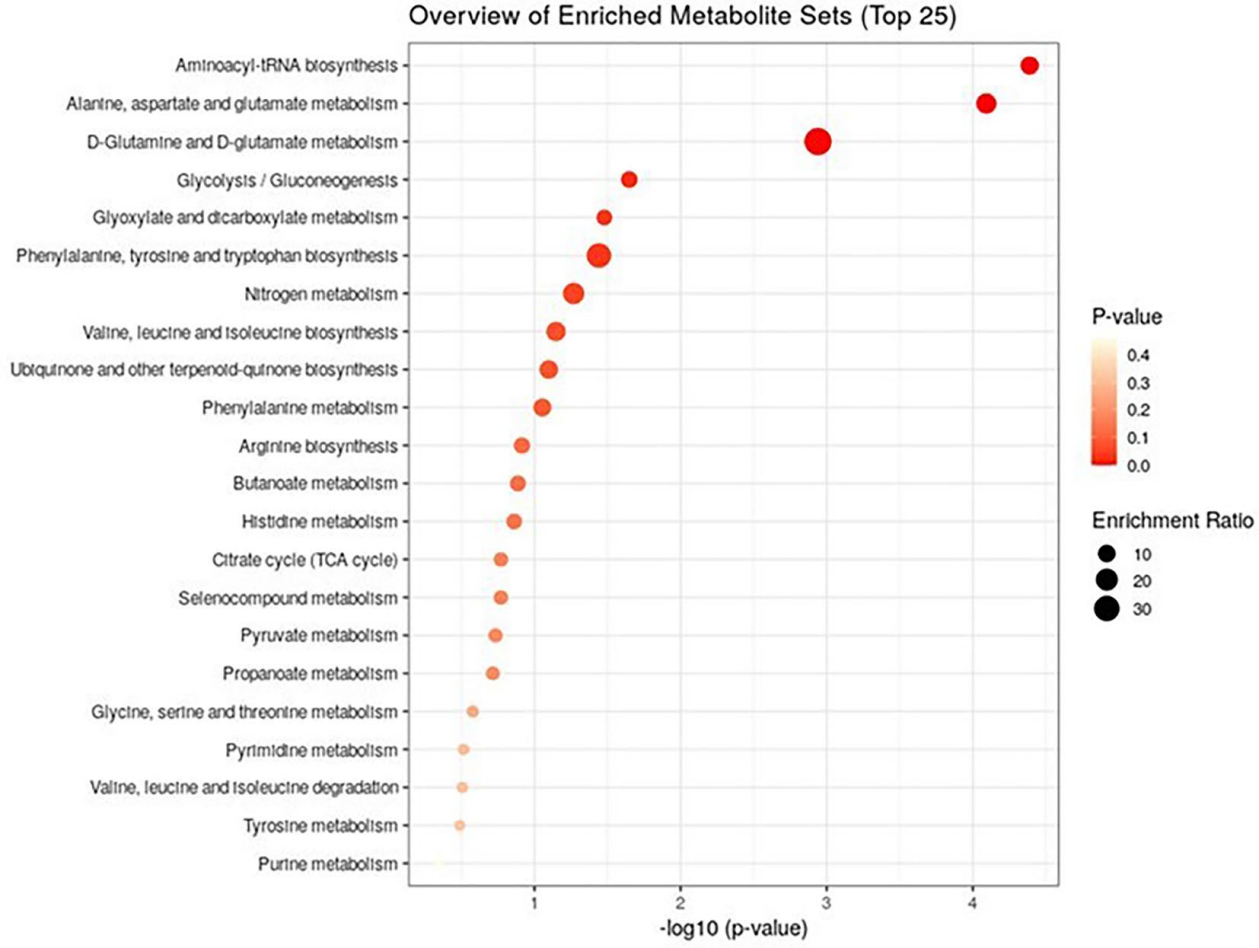
Dot plot of metabolic pathway influenced by statistically significant metabolites in hyperketonemic ewes or group HYK (n = 18; BHB ≥ 0.80 mmol/L). Color gradient and symbol size represent significant metabolite changes in the corresponding pathway.

Over representation analysis of the significant metabolites was performed using “Enrichment Analysis” in MetaboAnalyst 5.0 software to define which metabolic pathways were affected in HYK. The identified metabolic pathways were presented graphically as a dot plot (Fig. 3). Although a total of 21 metabolic pathways were found to be associated with significant metabolites, only 3 metabolic pathways showed an alteration in HYK (Table 3): (i) aminoacyl-tRNA biosynthesis; (ii) alanine, aspartate and glutamate metabolism; and (iii) d-glutamine and d-glutamate metabolism.

**Table 3:** Metabolic pathways influenced by significative metabolites in hyperketonemic ewes (Group HYK; n = 18; BHB ≥ 0.80 mmol/L) with their *p*-value.

Metabolic pathways	Total^1^	Hits^2^	Metabolites^3^	*p-*value	Holm-Bonferroni corrected
Aminoacyl-tRNA biosynthesis	48	5	Asparagine, glutamine, alanine, threonine and tyrosine	< 0.0001	0.003
Alanine, aspartate and glutamate metabolism	28	4	Asparagine, alanine, glutamine and succinate	< 0.0001	0.007
d-Glutamine and d-glutamate metabolism	6	1	Glutamine	0.001	0.095
Glycolysis/gluconeogenesis	26	2	Ethanol and acetate	0.023	1.0
Glyoxylate and dicarboxylate metabolism	32	2	Acetate and glutamine	0.033	1.0
Phenylalanine, tyrosine and tryptophan biosynthesis	4	1	Tyrosine	0.036	1.0

^1^Total number of metabolites in the pathway based on the Kyoto Encyclopedia of Genes and Genomes (KEGG) database.

^2^Number of metabolites influenced the pathways in this experiment.

^3^Metabolites identify by KEGG database.

## Discussion

Increased nutrient demand and decreased intake during early lactation may results in NEB^17,18^. Because of this, fatty acids and amino acids are mobilized from adipose and muscle tissues, respectively, predisposing animals in early lactation to the risk of metabolic disease such as ketosis^5–7^. Subclinical ketosis is less studied in dairy ewes compared to dairy cows but is not less important because it can significantly affect animal welfare and farm profitability^1,8^. Metabolomic profile changes in blood, milk, and urine including changes in amino acids, lipids, organic acids, carbohydrates, amines, vitamins, and others were found in dairy cows with increasing BHB levels and are still being studied^10,19^. Further studies about the metabolomics profile of animals is of critical importance to improve the understanding of ketosis in small ruminants and to develop biomarkers for an early diagnosis^20,21^. Because of this, metabolomic analysis of serum in hyperketonemic ewes by ^1^H-NMR was conducted.

In this study, animals in early lactation with blood BHB ≥ 0.80 mmol/L were considered hyperketonemic. However, it should be taken into consideration that a specific cut-off is not established for lactating sheep, and therefore the cut-off value used during pregnancy was applied^9^. In addition, blood sampling was performed only once because the experimental design was cross-sectional and was performed from the jugular vein. According to Mahrt et al.^22^, a single measurement of BHB in constantly fed dairy cows allowed the same accuracy for ketosis diagnosis compared to repeated measure within a day in ketosis diagnosis. Considering the postpartum sampling time, the peak incidence of subclinical ketosis in dairy cattle was found at 5 DIM^23^. In addition, the concentration of BHB did not differ between sampling from the jugular and coccygeal veins^6,22^. It is also true that some of the different metabolites assessed in this study may be influenced by cranial tissue metabolism. Therefore, further investigations with metabolomics approach are necessary to assess the possible differences according to the sampling location.

The presence of hyperketonemia, hypoglycemia and uremia are consistent with an early subclinical ketosis status postpartum^24^. The HYK group had higher concentration of serum BHB and urea, and a lower concentration of glucose. The urea serum concentration was slightly over the physiological range for sheep (2.86–7.14 mmol/L), whereas glucose concentration was within the normal range (2.78–4.44 mmol/L), although glucose was reduced compared with Non-HYK^25^. The rPCA analysis is a multivariate statistical method used as an explanatory clustering technique to identify differences between groups metabolites^26^. Our rPCA revealed a clear difference between the serum metabolome of the groups. This suggest that the HYK ewes are metabolically distinct from Non-HYK ewes, even though the BHB concentration threshold for postpartum hyperketonemia is not well established. From this point of view, hyperketonemic ewes may be considered separately from healthy animals.

The mobilization of adipose tissue is related to triacylglycerols break down into its components, NEFA and glycerol, which are then released into the blood stream^6,27^. In our study, the similarity in NEFA and glycerol concentrations, may indicate that adipose tissue was equally mobilized between groups. However, the type of matrix used in this study must be taken into consideration as each sample type offers a different aspect of metabolism. In fact, serum represents a primary carrier that contains metabolites related to all organs in the body^28–30^. Therefore, the metabolites found in serum may result from uptake and cellular metabolism, and be utilized by other tissues than the mammary gland. It is possible that an initial increase in NEFA and glycerol may be used by mammary gland to synthetize milk fat, the increase in which is a common feature during ketosis^31–33^. The NEFA can also be partially oxidized to ketone bodies such as acetoacetate, BHB and acetone in hepatic tissue^34^. BHB and acetone were identified by metabolomic approach in our study and they showed an increase in the HYK group. They may also be derived from ketogenic amino acids (lysine, leucine and isoleucine)^14,35^ which did not changed in this study suggesting no differential use to produce ketone bodies.

Leucine, isoleucine, and valine are branched-chain amino acids (BCAAs) used for protein synthesis in muscle. Low concentration of BCAAs are positively related to alanine concentration, which is a glucogenic amino acid highly concentrate in muscle. Reduced concentration of BCAA and alanine are associated with muscle mobilization^36–38^. In our study, both alanine and valine showed lower levels in HYK suggesting a catabolic state of muscle tissue. Additionally, 3-methylhistidine comes from muscle protein breakdown and it is considered as a biomarker of protein mobilization; whereas lower level of tyrosine was suggested as an indicator of reduced muscle growth^39,40^. The greater level of 3-methylhistidine and the lower level of tyrosine further suggest the hypothesis that there was protein mobilization by muscle tissue in HYK. However, creatine and its breakdown product, creatinine, are related to total muscle mass^41,42^. Creatine concentration is related to subclinical ketosis in dairy cows and weight loss in different ewe breeds^40,43^. The absence of changes in their concentrations may suggest that the total muscle loss was not different in HYK ewes. Comparable results for lower alanine and tyrosine levels, and greater 3-methylhistidine level were found in dairy cows affected by ketosis in the studies of Sun et al.^14^ and Wang et al.^43^. In contrast, the same studies identified an increase in valine level during subclinical ketosis and then showed no differences between clinical ketosis and healthy animals. The difference in valine results may be due to the different species and cut-off of BHB.

An increase in serum methanol and ethanol concentrations in hyperketonemic ewes and has also been reported in hyperketonemic dairy cows^19^. Methanol is a metabolite related to methane, a gas produced during ruminal fermentation by microbial cells^44–46^. However, dimethylsulfone and formate showed similar concentrations between groups indicating that methane production likely was not affected^47^. Increase in methanol may also be related to an increase in ethanol concentration that inhibits the methanol’s utilization by microorganisms^48^. Ethanol can be derived from anaerobic fermentation by yeasts^49^. This alcohol is an agonist of GABA receptors, so it has a depressive effect. The major product of ethanol in hepatic tissue is acetate to provide energy^50^. Acetate is a volatile fatty acid (VFA) produced by ruminal fermentation that increases during ketosis^51^ as reported in the present study. Acetate is an important energy substrate when bound to coenzyme A to produce acetyl-CoA and enters the TCA cycle^15^. Moreover, it may be used in brain metabolism, specifically in glial cells, potentially causing mitochondrial permeability and excitotoxic neuronal death^50^. Acetyl-CoA can also be derived from 2,3-butanediol^52^, a ruminal and intestinal microbial product that was increased in the HYK group. Another VFA is propionate^51,53^ which was not identified in our study, although the 3-hydroxyisobutyrate may be made from propionate and was increased in HYK. The above changes may suggest an alteration in ruminal fermentations in hyperketonemic ewes with potential relationships with the pathogenesis and symptoms of ketosis although further studies focused on sheep susceptibility would be needed. Except for dimethylsulfone and formate, similar results were found in the study by Lisuzzo et al.^19^ on subclinical ketosis in dairy cows.

Myo-inositol is a stereoisomeric form of inositol and represents an insulin mimetic metabolite because it promotes adipose tissue lipid storage and limits lipolysis rate^54^. Choline supports the transport of fatty acids, increases their oxidation and reduces the risk of hepatic lipidosis^14,55^. Choline can be converted in trimethylamine-*N*-oxide (TMAO), a marker of oxidative stress because it may affect energy metabolism^56–58^. Another metabolite related to β-oxidation is allantoin, a product of uric acid. Uric acid is related to triglycerides metabolism and its increase may limit enzymatic activity for their catabolism^43^. The analogous concentration between groups of myo-inositol, choline, TMAO, and allantoin may suggest that the β-oxidation of fatty acids was not influenced in our hyperketonemic ewes. Although subclinical ketosis is associated with a greater and altered lipid metabolism, the HYK ewes did not show these characteristics. As previously mentioned, a possible increment of NEFA may utilized by the mammary gland, leading to a lack of an increase in fatty acids oxidation.

The TCA cycle begins with the condensation of between acetyl-CoA and oxaloacetate. Acetyl-CoA may derive from fatty acid catabolism or pyruvate oxidation. Pyruvate may derive from amino acids (glycine, serine and alanine)^59^ among them only the alanine reduction in group HYK was significant. Alanine represents one of the major resources for gluconeogenesis, therefore it affects carbohydrate metabolism. It is known to be decreased during ketosis and fatty liver in dairy cows^60,61^. Pyruvate and glucose did not change in HYK ewes in the current study. Indeed, pyruvate can be used for gluconeogenesis to produce glucose^62^. These findings suggest that glucose concentration may be related to pyruvate and lower concentration of pyruvate due to lack of its precursors (glycine, serine and alanine) may affect glycemia and the development of ketosis. Asparagine is one precursor of oxaloacetate^59^ and was lower in the hyperketonemic ewes in agreement with other studies^57^. In this study, oxaloacetate was not identified. However, the reduction of its precursor may indicate a greater demand of oxaloacetate and an alteration of the TCA cycle. The next intermediates of TCA are citrate and isocitrate, which are maintained in equilibrium in the cell^63^. Isocitrate is subsequently converted to α-ketoglutarate that may derive from glutamate. Histidine, proline, glutamine and arginine are all metabolites related to glutamate production^64,65^. Glutamine can be converted to glutamate and then pyrroline-5-carboxylate, which links TCA and urea cycle. Arginine is converted into ornithine and urea in the final phase of the urea cycle^65^. The lower concentrations of glutamine, glutamate, histidine and arginine may suggest an alteration of both these cycles. Lower levels of glutamate, glutamine, and arginine were also found in dairy cows affected by ketosis^14,19,43^. Succinate is the subsequent intermediate of TCA whose precursors are threonine and methionine^27^. In this study, threonine and methionine were lower in group HYK whereas succinate was greater in the same group suggesting the amino acids use to produce succinate and consequently oxaloacetate for gluconeogenesis. However, fumarate is the next intermediate of TCA and not showed difference between groups. This metabolite may be synthetized by phenylalanine and tyrosine^59^. As previously mentioned, tyrosine was lower in HYK as reported in other studies^10^. These results may indicate that there was a disturbance of succinate dehydrogenase function and fumarate was maintained by its precursor. Succinate dehydrogenase is the only enzyme involved in the TCA cycle and in the electron transport chain (ETC)^66^. An alteration in succinate dehydrogenase might suggest an influence on ETC in hyperketonemic ewes as well as in hyperketonemic dairy cows. In fact, a similar result in succinate and fumarate concentrations was recently identified in dairy cows affected by subclinical ketosis^19^. In addition, Swartz et al.^67^ and Garcìa-Roche et al.^68^ identified an alteration of hepatic mitochondrial function. In the first study caused by an alteration of enzymes’ subunits involved in the mitochondrial respiratory chain, whereas in the second study by a reduction in the maximum respiratory rate of complex I, and an alteration in the rate of mitochondrial proteins acetylation in cows with increased BHB levels.

Several TCA-related amino acids are also important for the immune function, inflammatory process, and oxidative stress. In fact, both threonine and glutamate play a role in the regulation of the immune system. In particular, glutamate is involved in the activation and proliferation of immune system cells, gene expression and production of cytokines and antibodies, and reduction of cellular oxides^13,69,70^. In addition, histidine show antioxidant and anti-inflammatory qualities for removing reactive oxygen species (ROS) generated by cells during acute inflammation and suppressing the expression of pro-inflammatory cytokines^71,72^. Also related to antioxidants are glycine, serine, and methionine. Glycine and serine are biosynthetically related and are important regulators of glutathione synthesis to manage oxidative stress^13^. Methionine is involved in protein synthesis, antioxidant production, and methyl group donation. One of the routes of synthesis of this amino acid involves the oxidation of choline to betaine, which can be used as a methyl donor to synthesize methionine. Choline production from methionine is relatively high in ruminants because of its degradation in the rumen^73^. In this study, glycine and serine did not differ between the two groups, while threonine, glutamate, histidine and methionine were reduced in the HYK group. These findings suggest a potential alteration of the inflammatory response, immune system functions and management of oxidative stress status. Furthermore, glutamate may act as a neuroactive ligand for glutamate receptor 1, with a consequent excitatory effect^42^. The reduction of glutamate may play an important role in nervous depression if ketosis develops.

The main function of aminoacyl-tRNA biosynthesis is to catalyze the aminoacylation of transfer RNAs (tRNAs) involved in protein synthesis, angiogenesis and immune regulation^74^. During Alanine, Aspartate, and Glutamate metabolism there is biosynthesis of some amino acids (alanine, aspartate, asparagine, glutamate, and glutamine) and intermediates of the TCA cycle (oxaloacetate, citrate, succinate, fumarate). Therefore, this metabolic pathway is related with lipid, carbohydrates, and amino acid metabolisms. Furthermore, in human patient this pathway can be involved in the pathogenesis of metabolic syndrome^75^. The d-Glutamine and d-Glutamate metabolism was linked to Alanine, Aspartate, and Glutamate metabolism and concerns the glutamine/glutamate cycling. However, it should be kept in consideration that a specific reference library was not selected for both the sheep and the matrix used (blood) due to the lack of such options within the software and which may have influenced the actual outcome of the analysis^76^. For these reasons, further studies are needed to fully evaluate the altered metabolic pathways in this species during hyperketonemia.

## Conclusions

Our study demonstrated that the ^1^H-NMR metabolomic approach allows detection of metabolic changes that occur during hyperketonemia that may be related to the pathogenesis of ketosis. Indeed, the metabolic state of the hyperketonemic ewes suggests an alteration of ruminal fermentation; mobilization of body reserves; alteration in carbohydrates and amino acids metabolism; a potential alteration in ETC; influence on urea synthesis; alteration in nervous system, inflammatory response, and immune cell function.

## Methods

All animal procedures were conducted according to Directive 2010/63/EU of the European Parliament and the Council of the 22nd September 2010 on the protection of animals used for scientific purposes (Article 1, Paragraph 1, Letter b) and Italian legislation (D. Lgs. n. 26/2014, Article 2, Paragraph 1, Letter b). The study received the approval of the Ethics Committee of Sassari University (Protocol number 128469/2019) and was carried out in compliance with the ARRIVE guidelines. Informed consent was obtained from the owners for handling the animals and for clinical activities at the Veterinary Teaching Hospital, University of Padua.

### Animals, experimental design, and blood analysis

A cross-sectional experimental design was used. The experiment was conducted on 46 adult ewes, selected within 10 days of parturition from a flock located at a commercial farm in North Sardinia (Italy). The animals enrolled were the same subjects the previous work of Fiore et al.^77^ All enrolled animals lambed twins and there were 11 primiparous, 11 s parity, 10 third parity, and 14 with four or more parities. They were fed a total mixed ratio (TMR) formulated for lactating sheep (40–50 kg of body weight—BW) that contained 15% crude protein content and had a metabolizable energy value of 9.5 ME Mj/kg DM. The TMR contained 700 g of haylage, 400 g of hay, 200 g of silage maize, 150 g of soya, 150 g of flaked corn and 150 g of beet pulp. Furthermore, the ewes grazed natural pasture for 1 h/day. Animals were milked twice a day to calculate daily production from lambing through the first 30 days in milk (DIM). Body condition score (BCS) was rated on a scale of 1 to 5 points, with 1 being emaciated and 5 being extremely fat^78^, on the same day as clinical examination and blood sampling in each subject. Age and parity were also considered among characteristics of the ewes. Biological samples were collected from clinical healthy ewes that were examined by the Veterinarian of the University of Sassari (Italy). Animals that showed clinical signs of postpartum disease at or before the time of sampling were excluded from the study, as well as animals that suffered of OPT during pregnancy.

Blood samples were collected at 4.48 ± 0.94 DIM from the jugular vein using vacuum tubes containing a clot activator (9 mL; Terumo Venosafe, Leuvel, Belgium) as described in the study of Fiore et al.^77^. The blood was refrigerated at 4 °C, transported within 1 h at the same temperature to the laboratory of the University of Sassari (Italy)centrifuged immediately upon arrival at 3000 rpm × 10 min (Hettich® EBA 20 centrifuge, Stuttgart, DE, Germany). Two aliquots of serum were extracted and immediately sent on dry ice to the Department of Animal Medicine, Production and Health (MAPS) at the University of Padua (Italy) arrived within 24 h. One aliquot of the serum was stored at − 20 °C for the biochemical analysis and the other was stored at − 80 °C for the metabolomic analysis by ^1^H-NMR.

Serum biochemistry was performed in the laboratory of the Experimental Zooprophylactic Institute of Umbria and Marche (IZSUM, Perugia, Italy) as described in the study of Fiore et al.^77^.

All animals were divided in two groups based on serum BHB concentration. The Non-HYK or healthy group enrolled 28 ewes with a blood BHB < 0.80 mmol/L and the HYK or hyperketonemic group enrolled 18 ewes with a blood BHB ≥ 0.80 mmol/L^3,9^. Clinical data and parameters of the two groups were shown in Table 1.

### Metabolomic analysis

An NMR analysis solution was created with 10 mM 3-(trimethylsilyl)-propionic-2,2,3,3-d4 acid sodium salt (TSP) in D_2_O adjusted to pH 7.00 ± 0.02 using 1 M phosphate buffer containing 2 mM NaN_3_. TSP was used as an NMR chemical-shift reference, while NaN_3_ inhibited microbial proliferation as suggested by Zhu et al.^79^. Serum samples processed for ^1^H-NMR by thawing and centrifuging 1 mL of each sample for 15 min at 18,630×*g* at 4 °C. After centrifugation, 700 μL of supernatant were added to 100 μL of NMR analysis solution. Finally, each sample was centrifuged again for 15 min at 18,630×*g* at 4 °C.

^1^H-NMR spectra were recorded at 298 K with an AVANCE III spectrometer (Bruker, Milan, Italy) operating at a frequency of 600.13 MHz and equipped with Topspin 3.5 software. Following Zhu et al.^80^. the signals from broad resonances originating from large molecules were suppressed by a CPMG filter comprised of 400 echoes with a τ of 400 μs and a 180° pulse of 24 μs, for a total filter of 330 ms. The HOD residual signal was suppressed by means of presaturation. This was done by employing the cpmgpr1d sequence, part of the standard pulse sequence library. Each spectrum was acquired by summing 256 transients, separated by a recycle delay of 5 s, using 32 K data points over a 7184 Hz spectral window, with an acquisition time of 2.28 s.

The spectral phase was manually adjusted in Topspin, while the subsequent adjustments were performed in R computational language by means of a script developed in-house^81^. After the removal of the residual water signal, the^1^H-NMR spectra were baseline-corrected by means of peak detection, according to the “rolling ball” principle^82^, implemented in the baseline R package^83^. The signals were assigned by comparing, by means of Chenomx software (Chenomx Inc., Canada, ver. 8.3), their chemical shift and multiplicity with the Chenomx internal library (ver. 10) and with HMDB (http://hmdb.ca) library implemented in Chenomx (ver. 2) according to the Metabolomics standard initiative (MSI) for metabolites annotation^84^. This provided level 1, this allowed the confidence in the identification of each metabolite of the compounds in the first serum sample analyzed were quantified using an external standard using the principle of reciprocity^85^. The other samples were then normalized to the first by probabilistic quotient normalization, compensating for potential differences in water content. Quantification was performed by means of rectangular integration, considering one of the corresponding signals, free from interferences^86^. The recycle delay selected was five times longer than the longitudinal relaxation time of the protons employed for quantification, thus recovering 99.3% of their equilibrium magnetization^87^.

### Statistical analysis

Animal data and biochemical parameters were analyzed using S.A.S. version 9.4 (SAS Institute Inc., Cary, North Carolina, USA); whereas statistical analysis of metabolites obtained by ^1^H-NMR was conducted in R ver. 4.0.3 (R core team, Vienna, Austria)^81^. Data distribution was assessed using Shapiro normality test with a *p*-value ≤ 0.05 indicated a non-normal distribution. The metabolites that were not-normally distributed were transformed according to Box and Cox^88^. Based on the normal distribution and the equality of variance assessed by Levene test, animal data and biochemical parameters were analyzed by one-way ANOVA testing the effect of the group. The concentration of serum metabolites was evaluated by t-test for unpaired samples. A post-hoc pairwise comparison among means were performed using Bonferroni correction. In general, a significance limit (*p*-value) ≤ 0.05 was accepted, while a *p*-value between 0.05 and 0.1 were considered as trend to significance.

A robust principal component analysis (rPCA) model^89^ was built through the PcaHubert algorithm, implemented in the “rrcov” package of R software to summarize the structure of the data. First, the algorithm detects outlying samples, by computing their distance from the others along and orthogonally to the PCA plane. The optimal number of principal components (PCs) is finally determined. The rPCA model is summarized by a score-plot and a correlation plot. The score-plot highlights the overall structure of the data, by showing the samples in the PC space. The second plot highlights the molecules that mostly determine the structure of the data, showing the correlations between the concentration of each molecule and the PCs.

MetaboAnalyst 5.0 software (www.metaboanalyst.ca/MetaboAnalysts) is a comprehensive web-based tool designed to help users easily perform metabolomic data analysis, visualization, and functional interpretation^90^. The software function “Enrichment analysis” was used to perform an over representation analysis (ORA) on significant metabolites and used the compound name as Input type and Metabolites as Feature type to determine which metabolic pathways were associated with greater serum BHB concentration. Furthermore, the reference library selected was the pathway based, specifically the KEGG (84 metabolites based on human metabolic pathways) considering that no other species were available as reference library. Through MetaboAnalyst software, the website of PubChem (https://pubchem.ncbi.nlm.nih.gov/), Human Metabolome Database (HMDB; https://hmdb.ca/metabolites/) and Kyoto Encyclopedia of Genes and Genomes (KEGG; https://www.genome.jp/kegg/) were consulted to understand metabolites functions.

## Data Availability

The data are available by sending an email to the corresponding author.

## References

[1] Pesántez-Pacheco JL (2019). Influence of maternal factors (weight, body condition, parity, and pregnancy rank) on plasma metabolites of dairy ewes and their lambs. Animals.

[2] Doré V, Dubuc J, Bélanger AM, Buczinski S (2015). Definition of prepartum hyperketonemia in dairy goats. J. Dairy Sci..

[3] Rook JS (2000). Pregnancy toxemia of ewes, does, and beef cows. Vet. Clin. N. Am. Food Anim. Pract..

[4] Rezaei R, Wu Z, Hou Y, Bazer FW, Wu G (2016). Amino acids and mammary gland development: Nutritional implications for milk production and neonatal growth. J. Anim. Sci. Biotechnol..

[5] Kokkonen T (2005). Effect of body fatness and glucogenic supplement on lipid and protein mobilization and plasma leptin in dairy cows. J. Dairy Sci..

[6] Guo J, Peters RR, Kohn RA (2007). Effect of a transition diet on production performance and metabolism in periparturient dairy cows. J. Dairy Sci..

[7] Grummer RR (1993). Etiology of lipid-related metabolic disorders in periparturient dairy cows. J. Dairy Sci..

[8] Marutsova V, Marutsov P (2018). Subclinical and clinical ketosis in sheep-relationships between body condition scores and blood Β-hydroxybutyrate and non-esterified fatty acids concentrations. Tradit. Mod. Vet. Med..

[9] Panousis N (2018). Evaluation of a portable ketometer for on-site monitoring of blood β-hydroxybutyrate concentrations in dairy sheep and goats. Rev. Med. Vet. (Toulouse).

[10] Zhang G, Ametaj BN (2020). Ketosis an old story under a new approach. Dairy.

[11] Couperus AM (2021). Longitudinal metabolic biomarker profile of hyperketonemic cows from dry-off to peak lactation and identification of prognostic classifiers. Animals.

[12] Cainzos JM, Andreu-vazquez C, Guadagnini M, Rijpert-duvivier A (2022). A systematic review of the cost of ketosis in dairy cattle. J. Dairy Sci..

[13] Zhang G (2017). Metabotyping reveals distinct metabolic alterations in ketotic cows and identifies early predictive serum biomarkers for the risk of disease. Metabolomics.

[14] Sun LW (2014). 1H-nuclear magnetic resonance-based plasma metabolic profiling of dairy cows with clinical and subclinical ketosis. J. Dairy Sci..

[15] Sun L (2017). Metabolic profiling of stages of healthy pregnancy in Hu sheep using nuclear magnetic resonance (NMR). Theriogenology.

[16] Cullen CH, Ray GJ, Szabo CM (2013). A comparison of quantitative nuclear magnetic resonance methods: Internal, external, and electronic referencing. Magn. Reson. Chem..

[17] Fiore E (2017). Hepatic lipidosis in high yielding dairy cows during the transition period: Haematochemical and histopathological findings. Anim. Prod. Sci..

[18] Drackley JK (1999). Biology of dairy cows during the transition period: The final frontier?. J. Dairy Sci..

[19] Lisuzzo A (2022). Differences in the serum metabolome profile of dairy cows according to the BHB concentration revealed by proton nuclear magnetic resonance spectroscopy (1 H-NMR). Sci. Rep..

[20] Kenéz Á, Dänicke S, Rolle-Kampczyk U, von Bergen M, Huber K (2016). A metabolomics approach to characterize phenotypes of metabolic transition from late pregnancy to early lactation in dairy cows. Metabolomics.

[21] Singh B, Mal G, Gautam SK, Mukesh M, Singh B (2019). Metabolomics in livestock sciences. Advances in Animal Biotechnology.

[22] Mahrt A, Burfeind O, Heuwieser W (2014). Effects of time and sampling location on concentrations of β-hydroxybutyric acid in dairy cows. J. Dairy Sci..

[23] McArt JAA, Nydam DV, Oetzel GR (2012). Epidemiology of subclinical ketosis in early lactation dairy cattle. J. Dairy Sci..

[24] Moghaddam G, Hassanpour A (2008). Comparison of blood serum glucose, beta hydroxybutyric acid, blood urea nitrogen and calcium concentrations in pregnant and lambed ewes. J. Anim. Vet. Adv..

[25] Kaneko JJ, Hervey JW, Bruss ML (2008). Clinical Biochemistry of Domestic Animals.

[26] Sundekilde U, Larsen L, Bertram H (2013). NMR-based milk metabolomics. Metabolites.

[27] Xue Y, Guo C, Hu F, Liu J, Mao S (2018). Hepatic metabolic profile reveals the adaptive mechanisms of ewes to severe undernutrition during late gestation. Metabolites.

[28] Goldansaz SA (2017). Livestock metabolomics and the livestock metabolome: A systematic review. PLoS ONE.

[29] Kirwan J (2013). Metabolomics for the practising vet. In Pract..

[30] Zhang A, Sun H, Wang X (2012). Serum metabolomics as a novel diagnostic approach for disease: A systematic review. Anal. Bioanal. Chem..

[31] Sun X (2019). High expression of cell death-inducing DFFA-like effector a (CIDEA) promotes milk fat content in dairy cows with clinical ketosis. J. Dairy Sci..

[32] Gross J, van Dorland HA, Bruckmaier RM, Schwarz FJ (2011). Performance and metabolic profile of dairy cows during a lactational and deliberately induced negative energy balance with subsequent realimentation. J. Dairy Sci..

[33] Buttchereit N, Stamer E, Junge W, Thaller G (2010). Evaluation of five lactation curve models fitted for fat:protein ratio of milk and daily energy balance. J. Dairy Sci..

[34] Pereira RA (2013). Metabolic parameters and dry matter intake of ewes treated with butaphosphan and cyanocobalamin in the early postpartum period. Small Rumin. Res..

[35] Yudkoff M (2005). Response of brain amino acid metabolism to ketosis. Neurochem. Int..

[36] Appuhamy JADRN, Knapp JR, Becvar O, Escobar J, Hanigan MD (2011). Effects of jugular-infused lysine, methionine, and branched-chain amino acids on milk protein synthesis in high-producing dairy cows. J. Dairy Sci..

[37] Liu S (2021). Isoleucine increases muscle mass through promoting myogenesis and intramyocellular fat deposition. Food Funct..

[38] Ribeiro DM (2019). Amino acid profiles of muscle and liver tissues of Australian Merino, Damara and Dorper lambs under restricted feeding. J. Anim. Physiol. Anim. Nutr. (Berl.).

[39] Houweling M, van der Drift SGA, Jorritsma R, Tielens AGM (2012). Technical note: Quantification of plasma 1- and 3-methylhistidine in dairy cows by high-performance liquid chromatography-tandem mass spectrometry. J. Dairy Sci..

[40] Palma M (2016). The hepatic and skeletal muscle ovine metabolomes as affected by weight loss: A study in three sheep breeds using NMR-metabolomics. Sci. Rep..

[41] Megahed AA, Hiew MWH, Ragland D, Constable PD (2019). Changes in skeletal muscle thickness and echogenicity and plasma creatinine concentration as indicators of protein and intramuscular fat mobilization in periparturient dairy cows. J. Dairy Sci..

[42] Kohlmeier M, Kohlmeier M (2015). Amino acids and nitrogen compounds. Nutrient Metabolism.

[43] Wang Y (2016). Pathway analysis of plasma different metabolites for dairy cow ketosis. Ital. J. Anim. Sci..

[44] Goopy JP (2014). Low-methane yield sheep have smaller rumens and shorter rumen retention time. Br. J. Nutr..

[45] Lawton TJ, Ham J, Tianlin S, Rosenzweig AC (2014). Structural conservation of the B subunit in the ammonia monooxygenase/particulate methane monooxygenase superfamily. Proteins.

[46] Auffret MD (2018). Identification, comparison, and validation of robust rumen microbial biomarkers for methane emissions using diverse Bos Taurus breeds and basal diets. Front. Microbiol..

[47] Yanibada B (2020). Inhibition of enteric methanogenesis in dairy cows induces changes in plasma metabolome highlighting metabolic shifts and potential markers of emission. Sci. Rep..

[48] Vantcheva ZM, Pradhan K, Hemken RW (1970). Rumen methanol in vivo and in vitro. J. Dairy Sci..

[49] Hungate RE, Hungate RE (1966). Chapter II—The rumen bacteria. The Rumen and its Microbes.

[50] Pawlosky RJ (2010). Alterations in brain glucose utilization accompanying elevations in blood ethanol and acetate concentrations in the rat. Alcohol. Clin. Exp. Res..

[51] Pechová A, Nečasová A (2018). The relationship between subclinical ketosis and ruminal dysfunction in dairy cows. Ann. Anim. Sci..

[52] Mathison GW, Fenton M, Milligan LP (1981). Utilization of 2,3-butanediol by sheep. J. Anim. Sci..

[53] Basoglu A, Baspinar N, Tenori L, Licari C, Gulersoy E (2020). Nuclear magnetic resonance (NMR)-based metabolome profile evaluation in dairy cows with and without displaced abomasum. Vet. Q..

[54] Kim JN, Han SN, Kim HK (2014). Phytic acid and myo-inositol support adipocyte differentiation and improve insulin sensitivity in 3T3-L1 cells. Nutr. Res..

[55] Gao H (2008). Metabonomic profiling of renal cell carcinoma: High-resolution proton nuclear magnetic resonance spectroscopy of human serum with multivariate data analysis. Anal. Chim. Acta.

[56] Makrecka-Kuka M (2017). Trimethylamine N-oxide impairs pyruvate and fatty acid oxidation in cardiac mitochondria. Toxicol. Lett..

[57] Xu C (2016). 1H-nuclear magnetic resonance-based plasma metabolic profiling of dairy cows with fatty liver. Asian-Australas. J. Anim. Sci..

[58] Zhao XJ (2011). Dynamic metabolic response of mice to acute mequindox exposure. J. Proteome Res..

[59] Nelson DL, Cox MM (2006). Principi di Biochimica di Lehninger. Lehninger Principles of Biochemistry.

[60] Luke TDW, Pryce JE, Wales WJ, Rochfort SJ (2020). A tale of two biomarkers: Untargeted 1H NMR metabolomic fingerprinting of BHBA and NEFA in early lactation dairy cows. Metabolites.

[61] Klein MS (2013). Correlations between milk and plasma levels of amino and carboxylic acids in dairy cows. J. Proteome Res..

[62] Sun HZ (2017). Lactation-related metabolic mechanism investigated based on mammary gland metabolomics and 4 biofluids’ metabolomics relationships in dairy cows. BMC Genomics.

[63] Garnsworthy PC, Masson LL, Lock AL, Mottram TT (2006). Variation of milk citrate with stage of lactation and de novo fatty acid synthesis in dairy cows. J. Dairy Sci..

[64] Qi SW (2012). H NMR-based serum metabolic profiling in compensated and decompensated cirrhosis. World J. Gastroenterol..

[65] Albaugh VL, Mukherjee K, Barbul A (2017). Proline precursors and collagen synthesis: Biochemical challenges of nutrient supplementation and wound healing. J. Nutr..

[66] Martínez-Reyes I, Chandel NS (2020). Mitochondrial TCA cycle metabolites control physiology and disease. Nat. Commun..

[67] Swartz TH (2021). Characterization of the liver proteome in dairy cows experiencing negative energy balance at early lactation. J. Proteomics.

[68] García-Roche M (2019). Impaired hepatic mitochondrial function during early lactation in dairy cows: Association with protein lysine acetylation. PLoS ONE.

[69] Duval D, Demangel C, Munier-Jolain K, Miossec S, Geahel I (1991). Factors controlling cell proliferation and antibody production in mouse hybridoma cells: I. Influence of the amino acid supply. Biotechnol. Bioeng..

[70] Li P, Yin YL, Li D, Kim WS, Wu G (2007). Amino acids and immune function. Br. J. Nutr..

[71] Peterson JW, Boldogh I, Popov VL, Saini SS, Chopra AK (1998). Anti-inflammatory and antisecretory potential of histidine in Salmonella-challenged mouse small intestine. Lab. Investig..

[72] Feng RN (2013). Histidine supplementation improves insulin resistance through suppressed inflammation in obese women with the metabolic syndrome: A randomised controlled trial. Diabetologia.

[73] Coleman DN (2019). Hepatic betaine-homocysteine methyltransferase and methionine synthase activity and intermediates of the methionine cycle are altered by choline supply during negative energy balance in Holstein cows. J. Dairy Sci..

[74] Nie A, Sun B, Fu Z, Yu D (2019). Roles of aminoacyl-tRNA synthetases in immune regulation and immune diseases. Cell Death Dis..

[75] Sookoian S, Pirola CJ (2012). Alanine and aspartate aminotransferase and glutamine-cycling pathway: Their roles in pathogenesis of metabolic syndrome. World J. Gastroenterol..

[76] Wieder C (2021). Pathway analysis in metabolomics: Recommendations for the use of over-representation analysis. PLoS Comput. Biol..

[77] Fiore E (2021). Milk fatty acids composition changes according to β-hydroxybutyrate concentrations in ewes during early lactation. Animals.

[78] Russel A (1984). Body condition scoring of sheep. In Pract..

[79] Zhu C (2020). First steps toward the giant panda metabolome database: Untargeted metabolomics of feces, urine, serum, and saliva by 1H NMR. J. Proteome Res..

[80] Zhu C, Faillace V, Laus F, Bazzano M, Laghi L (2018). Characterization of trotter horses urine metabolome by means of proton nuclear magnetic resonance spectroscopy. Metabolomics.

[81] Colin B (2018). Using Boosted Regression Trees and Remotely Sensed Data to Drive Decision-Making. Open J. Stat..

[82] Kneen MA, Annegarn HJ (1996). Algorithm for fitting XRF, SEM and PIXE X-ray spectra backgrounds. Nucl. Instrum. Methods Phys. Res. Sect. B Beam Interact. with Mater. Atoms.

[83] Liland KH, Almøy T, Mevik BH (2010). Optimal choice of baseline correction for multivariate calibration of spectra. Appl. Spectrosc..

[84] Salek RM, Steinbeck C, Viant MR, Goodacre R, Dunn WB (2013). The role of reporting standards for metabolite annotation and identification in metabolomic studies. Gigascience.

[85] Hoult DI (2011). The principle of reciprocity. J. Magn. Reson..

[86] Foschi C (2018). Urine metabolome in women with *Chlamydia trachomatis* infection. PLoS ONE.

[87] Schönberger, T. *Guideline for qNMR Analysis*, 1–24 (2019).

[88] Box GEP, Cox DR (1964). An analysis of transformations. J. R. Stat. Soc. Ser. B.

[89] Hubert M, Rousseeuw PJ, Van den Branden K (2005). ROBPCA: A new approach to robust principal component analysis. Technometrics.

[90] Chong J (2018). MetaboAnalyst 4.0: Towards more transparent and integrative metabolomics analysis. Nucleic Acids Res..

